# Integrating social determinants into routine dental care for a person‐centered approach

**DOI:** 10.1002/jdd.13794

**Published:** 2024-12-04

**Authors:** Jill York, Shawn Kelly, Cheila Garcia

**Affiliations:** ^1^ Department of Community Health, Rutgers School of Dental Medicine Newark New Jersey USA; ^2^ Department of Restorative Dentistry, Rutgers School of Dental Medicine Newark New Jersey USA; ^3^ Extramural Clinics at Rutgers School of Dental Medicine Newark New Jersey USA

**Keywords:** comprehensive care, dental health, person‐centered care, social determinants of health, HIV/AIDS

## 1 PROBLEM

Social determinants of health (SDOH) are the conditions that influence individuals throughout life, including economic stability, access to education and healthcare, neighborhood environments, and social support.^1^ Research indicates that SDOH impacts up to 50% of health outcomes, while healthcare access contributes 16% and health behaviors account for 34%.^2^ Improving SDOH may enhance engagement in care by reducing barriers, which can facilitate better health management. Addressing and collecting data on SDOH is essential for providing comprehensive, person‐centered care.

## 2 SOLUTION

**FIGURE 1:**
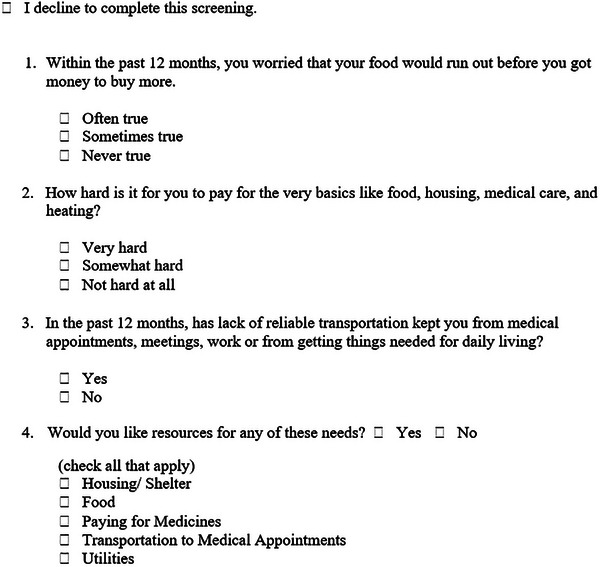
Social needs screening tool.

Funded by the Health Resources and Services Administration, Rutgers School of Dental Medicine (RSDM) is one of 12 Community Based Dental Partnership Programs whose goal is to improve oral health access for low‐income people with HIV (PWH) and train dental students. RSDM partnered with the Northeast/Caribbean AIDS Education Training Center for a Practice Transformation Project (PTP) to conduct a SDOH screening on 60% of PWH attending comprehensive/periodic visits between April 1 and June 30, 2024. RSDM developed a four‐question screening tool (Figure 1) focusing on financial resource strain, food insecurity, and transportation challenges. The instrument is adapted from evidence‐based material such as PREPARE and the Accountable Health Communities Tool.^3^ Additionally, a guide was developed to provide information on support services for those in need.

## 3 RESULTS

**FIGURE 2:**
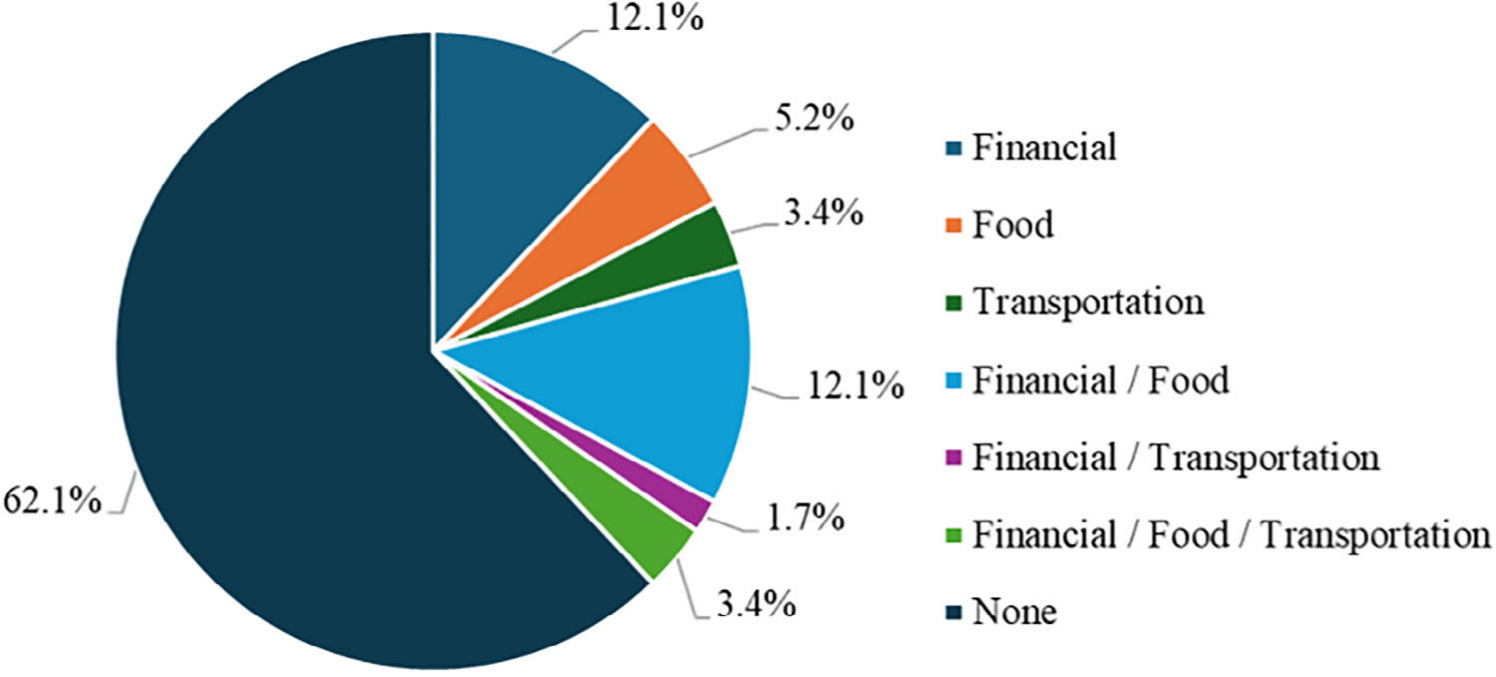
Social needs of the respondents.

During the project period, 70 PWH were seen in the clinic. Among them, 62 (88.6%) received the screening assessment, 58 (82.9%) completed it, 22 (37.9%) requested and received information about available social/support services, and nearly half (45.5%) identified multiple resource needs (Figure 2). Specifically, 17 (77.3%) reported financial resource strain, 12 (54.5%) experienced food instability, and five (22.7%) faced transportation uncertainty (Figure 2).

These findings highlight the social and financial barriers people encounter when seeking care. Screening for social needs during routine dental care presents several challenges affecting patient engagement and support provided. A major concern is that the SDOH survey often addresses socially stigmatizing topics that can make people uncomfortable or feel judged. Additionally, individuals may have multiple, intersecting social needs, making it difficult to prioritize and address. Furthermore, it is difficult to maintain an up‐to‐date resource guide amid ongoing changes in funding, program availability, and service capacities. Addressing these challenges requires a multi‐faceted approach that includes provider education, system‐level changes, and a focus on building trust and rapport with patients.

Screening for social needs has yielded several key insights that can improve the support provided and outcomes. First, maintaining confidentiality is essential. Ensuring patients feel safe and secure when sharing sensitive information is crucial for accurate assessments. Second, clear, and empathetic communication is vital. Patients need to understand the purpose of screening, how it benefits their care, and their involvement in the process. Finally, social needs are dynamic and may evolve. Regular and ongoing screening is crucial for adapting to changing circumstances and providing timely support. Piloted with PWH, this PTP reveals broader social and economic factors impacting individuals in the dental setting, underscoring the importance of a patient‐centered approach to addressing the social needs of all patients.

## CONFLICT OF INTEREST STATEMENT

The authors declare no conflicts of interest.
